# Contemporary Outcomes of Degenerative Mitral Valve Surgery in a Regional Tertiary Care Center

**DOI:** 10.3390/jcm13226751

**Published:** 2024-11-09

**Authors:** Paolo Berretta, Michele Galeazzi, Francesca Spagnolo, Martina Giusti, Simone D’Alessio, Olimpia Bifulco, Emanuele Di Campli, Francesca Mazzocca, Pietro Giorgio Malvindi, Carlo Zingaro, Alessandro D’Alfonso, Marco Di Eusanio

**Affiliations:** Cardiac Surgery Unit, Lancisi Cardiovascular Center, Polytechnic University of Marche, 60121 Ancona, Italy; p.berretta@icloud.com (P.B.); michelegaleazzi96@gmail.com (M.G.); francesca.spagnolo@ospedaliriuniti.marche.it (F.S.); martina.giusti@ospedaliriuniti.marche.it (M.G.); simone.dalessio@ospedaliriuniti.marche.it (S.D.); emanuele.dicampli@ospedaliriuniti.marche.it (E.D.C.); francesca.mazzocca@outlook.it (F.M.); p.g.malvindi@staff.univpm.it (P.G.M.); carlo.zingaro@ospedaliriuniti.marche.it (C.Z.); alessandro.dalfonso@ospedaliriuniti.marche.it (A.D.)

**Keywords:** mitral surgery, mitral valve repair, degenerative mitral valve disease, minimally invasive mitral valve surgery, transaxillary mitral valve surgery

## Abstract

**Objective**: As percutaneous mitral valve techniques become more prevalent, it is important to evaluate the contemporary outcomes of surgical mitral valve interventions. This study assessed the current results and procedural trends of mitral valve surgery for degenerative mitral regurgitation (DMR) at a regional tertiary care center. **Methods**: Data were analyzed from 693 consecutive DMR patients who underwent isolated mitral valve operations, with or without tricuspid valve repair and atrial fibrillation ablation between 2017 and 2024. The outcomes were defined according to MVARC criteria. The study endpoints included successful mitral valve repair, in-hospital results, and operative and long-term mortality. Logistic regression was applied to assess the impact of valve lesions and patient risk factors on the probability of valve repair. Survival was analyzed using Kaplan–Meier methodology. The follow up was 100% complete. **Results**: Mitral valve repair was performed in 90.9% of cases, with only 0.9% requiring the conversion to replacement due to unsuccessful repair. Posterior leaflet lesions had the highest success rate (93.4%), while anterior leaflet lesions had a lower rate (86.2%), with anterior pathology being a negative predictor of repair (OR 2.57, *p* = 0.02). The type of lesion (prolapse vs. flail), the commissural involvement, and the increased risk for SAM had no statistically significant impact on valve repair outcome. Less invasive transaxillary access was used in 63.2% of patients, and its adoption increased significantly (from 50.9% to 67.4% *p* = 0.03) over time, resulting in more frequent fast-track extubation and home discharges. The rate of in-hospital mortality was 0.6%, while the rate of 5-year survival was 95.5%. **Conclusions**: Contemporary surgical techniques for DMR lead to high repair rates and excellent recovery outcomes. Despite the rise in transcatheter options, our findings confirm that surgery remains the gold standard for most DMR patients.

## 1. Introduction

Degenerative mitral valve regurgitation (DMR) is a widespread and clinically relevant condition in Western countries, contributing significantly to morbidity and healthcare costs [1]. Surgical intervention is the gold standard treatment for DMR patients, aiming to achieve durable valve repair and improve long-term outcomes. Recently, advances in surgical methods, including the adoption of less invasive approaches, have led to improved outcomes and reduced recovery times for patients undergoing DMR surgery [2,3,4,5]. However, in an era where transcatheter techniques have emerged as a promising alternative to surgery, the outcomes of contemporary surgical interventions need continuous benchmarking alongside these newer, less invasive options. In fact, as technologies and knowledge about mitral pathology, including perioperative care, advance, it is crucial for mitral surgery results to constantly be up to date to make a real and true comparison between these different approaches. This study analyzed contemporary outcomes of isolated mitral valve surgery for DMR at a regional tertiary care center, focusing on operative techniques, valve repair outcomes, procedural trends, and early and long-term results.

## 2. Materials and Methods

### 2.1. Study Population and Data Collection

The present study was a retrospective outcome evaluation from institutional records with prospective data entry. All patients with degenerative mitral valve regurgitation (DMR) who underwent isolated mitral valve surgery from January 2017 to May 2024 at Lancisi Cardiovascular Center in Ancona were included. The surgical approach included full sternotomy and less invasive transaxillary access [4]. DMR was defined as mitral regurgitation secondary to myxomatous disease [Barlow’s disease, fibroelastic deficiency (FED), and form fruste], or flail/prolapse leaflet [6]. Patients with no degenerative mitral valve disease (rheumatic, functional, endocarditis, and others), concomitant mitral valve stenosis, and those who had already undergone surgical or transcatheter procedures were excluded. Isolated mitral valve surgery was defined as mitral operation without concomitant coronary artery bypass graft surgery, aortic valve procedure, or root/ascending aorta replacement. Concomitant surgery for atrial fibrillation (AF), tricuspid valve repair, or patent foramen ovale closure was allowed. The patient demographics, medical history, operative and in-hospital outcomes, and echocardiographic data were collected at the time of each patient’s admission and during each subsequent follow-up visit. The data were coded according to the Mitral Valve Academy Research Consortium (MVARC) endpoint definitions and the current European Society of Cardiology or ACC/AHA/HRS guidelines and Euroscore II model [7,8,9]. Survival data were derived from the Marche regional administration records (completeness 100%). All the patients underwent preoperative echocardiographic examination [transthoracic and trans-oesophageal (TOE)] by the institutional core echocardiography laboratory. A pre-discharge transthoracic control was performed in all patients. The assessment of heart valve pathologies and their grading were in accordance with the current recommendations and guidelines. The study was approved by the local institutional review board (n. 2020189, 30 July 2020), and patients gave informed consent when required.

### 2.2. Study Outcomes

The study outcomes were successful mitral valve repair, operative and long-term mortality, and in-hospital results. The outcomes were defined according to MVARC endpoint definitions [8] and the EuroSCORE II model [9]. Successful mitral valve repair was defined as the absence of (1) mitral regurgitation > mild, (2) early reintervention (within 30 days), and (3) mitral valve prosthesis implantation. Operative mortality (according to ES II) was defined as death in the same hospital where the operation took place before discharge from the hospital. Stroke was defined as a duration of a focal or global neurological deficit ≥24 h, or <24 h if available neuroimaging documents show a new intracranial or subarachnoid hemorrhage, central nervous system infarction, or if the neurological deficit results in death. Low cardiac output was defined as the need for inotropic support >24 h, the use of an intra-aortic balloon pump (IABP), or extracorporeal membrane oxygenation ECMO. Myocardial infarction (MI) involves either periprocedural (≤48 h after the procedure) or spontaneous (>48 h after the procedure) MI according to the MVARC criteria [8]. Dialysis was defined as postoperative acute kidney injury requiring renal replacement therapy. MVARC’s technical success included the absence of procedural mortality, successful access, correct positioning of the first intended device, and freedom from emergency surgery or reintervention related to the device or access procedure.

### 2.3. Surgical Technique

All patients were operated on under general anesthesia, and TOE was performed before and during the operation to monitor and evaluate the procedural result. Our less invasive transaxillary approach has been previously described [4]. Briefly, with the patient in a supine position, a 4 to 5 cm skin incision was made in the right anterior axillary line at the level of the 3rd or 4th intercostal space. The femoral artery was cannulated for arterial inflow. Venous drainage was obtained by cannulation of the femoral and jugular veins. After the CPB institution, the pericardium was opened, and 7 stay sutures were placed. The ascending aorta was clamped through the same skin incision using CygnetVR Flexible Clamp (Vitalitec, Plymouth, MA, USA). Histidine–tryptophan–ketoglutarate (CustodiolVR) or del Nido cardioplegia was delivered in an antegrade fashion via the aortic root. The left atrium was opened using a left atrial atriotomy along the interatrial groove. The mitral valve apparatus was exposed to direct vision with the aid of an atrial retractor; no video assistance tool was used. Full sternotomy access involved a complete median sternotomy and cardiopulmonary bypass institution using ascending aorta and bicaval cannulation. The left side of the heart was vented through the right superior pulmonary vein. Antegrade infusion of histidine–tryptophan–ketoglutarate (CustodiolVR) or del Nido cardioplegia via the aortic root was used for myocardial protection. The left atrium is entered along the interatrial groove. The choice of surgical approach was made at the discretion of the operating surgeon. Exclusion criteria for transaxillary access were severe chest wall deformities or calcifications of the ascending aorta at the cross-clamping site. Mitral valve repair was performed using surgeons’ standardized techniques, regardless of the surgical access. It included chordal replacement with polytetrafluoroethylene chords or prefabricated loops, leaflet resection, sliding plasty, cleft/indentation closure, and edge-to-edge repair. A semi-rigid annuloplasty ring was used in all repair procedures. For mitral valve replacement, common stented biologic or mechanical substitutes were implanted using interrupted sutures with pledgets. Concomitant AF surgery included left atrial or bi-atrial ablation using radiofrequency or cryoablation in addition to the closure of the left atrial appendage. Patients with long-standing persistent atrial fibrillation and enlarged left atria were not deemed to be good candidates for rhythm correction therapy. Concomitant tricuspid valve repair with a semi-rigid ring was performed as indicated.

### 2.4. Statistical Analysis

Continuous variables were expressed as mean ± standard deviation (SD), and categorical variables as percentages. Where continuous variables did not follow a normal distribution, the median and interquartile range (IQR) were reported. In all cases, missing data were not defaulted to negative, and denominators reflect only cases reported. Comparisons of groups were performed using unpaired t-test or the Mann–Whitney U test (continuous variables) and chi-squared test (categorical variables). Trends across time groups were analyzed using Mantel–Haenszel tests of the trend for categorical variables or Kruskal–Wallis tests for continuous variables. The multivariable association between the type of valve lesion [prolapse, flail, site (PML vs. AML vs. Bilealfet), commissural prolapse/flail, annulus dilatation) and the probability of successful valve repair was assessed using multivariable logistic regression. The model was adjusted for potential confounders selected a priori based on their clinical significance that may directly influence the in-hospital results [age, sex, obesity, chronic lung disease, diabetes, atrial fibrillation, peripheral arteriopathy, cerebrovascular arteriopathy, pulmonary hypertension, dialysis, reduced left ventricular ejection fraction, NYHA III-IV, type of disease (FED vs. Barlow vs. form fruste) and increased risk for SAM]. The backward stepwise method was used to build the final model. The results are presented as adjusted odds ratios (OR) with a 95% confidence interval (CI). Multicollinearity was assessed using the variance inflation factor. Survival was evaluated with a life table and Kaplan–Meier analysis. The level of significance, α, was set at 5%. Statistical analysis was performed using Statistical Package for Social Sciences version 29.0 (IBM SPSS Inc., Chicago, IL, USA).

## 3. Results

### 3.1. Baseline Demographic and Echocardiographic Characteristics

**Figure 1:**
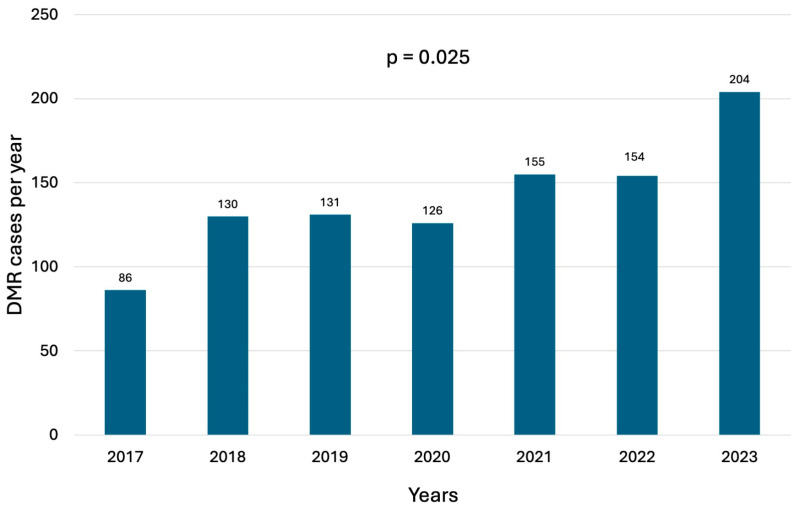
Number of annual surgical mitral valve procedures for DMR.

A total of 693 consecutive patients underwent mitral valve surgery for DMR during the study period, with a trend toward an increasing number of annual cases (Figure 1). Baseline characteristics are listed in Table 1. The median age was 66 years (IQR, 57–74 years), 35.1% (n = 244) of patients were women, and 32.9% (n = 219) of patients had NYHA class III or IV heart failure. Preoperative atrial fibrillation was observed in 23.9% of cases. The median Euroscore II was 2.4% (IQR 1–4.9). DMR was classified as FED in 43.1% of patients, Barlow’s disease in 28.7%, and form fruste in 28.1%. Preoperative echocardiography revealed that the majority of patients had posterior mitral leaflet (PML) involvement (62.6%), followed by bileaflet involvement (24%) and anterior mitral leaflet (AML) involvement (7.5%). Annular dilatation was highly prevalent, affecting 85.1% of patients. Commissural prolapse was identified in 6.7% of cases, and 37.6% of patients were at increased risk for systolic anterior motion (end-diastolic diameter < 45 mm, aorto-mitral angle < 120°, coaptation–septum distance < 25 mm, PML > 15 mm, AML/PML < 1 cm, basal septal diameter > 15 mm, A2 height differential > 5 mm, pre-repair SAM). Details of the echocardiographic findings are summarized in Table 2.

### 3.2. Operative Data and Procedural Outcomes

**Figure 2:**
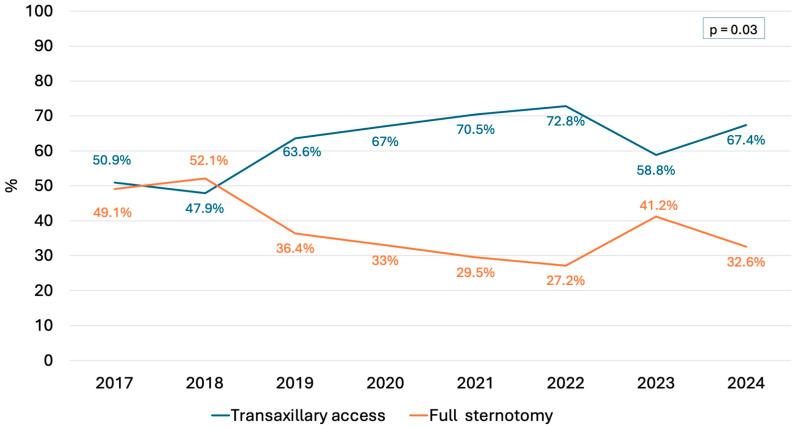
The trend in surgical approach.

Operative data are listed in Table 3. Surgical access was achieved through transaxillary minithoracotomy in 438 patients (63.2%) and full sternotomy in 255 (36.8%). Over the study period, there was a substantial increase in the use of less invasive transaxillary access (from 50.9% to 67.4%) and a decline in the use of conventional sternotomy (from 49.1.% to 32.6%, *p* = 0.03) (Figure 2). Mitral valve repair was performed in 90.9% of cases, while 8.1% required valve replacement, with 0.9% being converted to replacement due to unsuccessful repair. The likelihood of valve repair was significantly higher in patients with isolated PML (93.4%) and bileaflet (95.4%) prolapse/flail compared with those with isolated AML lesions (86.2%) (*p* = 0.04). After adjusting for potential confounders, AML lesions (OR 2.57, 95% CI 1.18–5.59, *p* = 0.02) were confirmed to be a negative predictor of successful mitral valve repair, as well as age (OR 1.05, 95% CI 1.03–1.08, *p* < 0.001). Conversely, the type of lesion (prolapse vs. flail), the commissural involvement, the increased risk for SAM, and the annulus dilatation had no impact on valve repair outcome (Table 4).

The most common leaflet repair technique was a chordal replacement (n = 364 54.1%) followed by resection (n = 119 17.7%), edge-to-edge (n = 90 13.4%), and sliding plasty (n = 32 4.8%). A second run of cardiopulmonary bypass was required in 27 patients (3.9%), 3 of which ultimately received mitral valve replacement. MVARC technical success was achieved in 97.5% of cases.

### 3.3. Early and Late Outcomes

**Figure 3:**
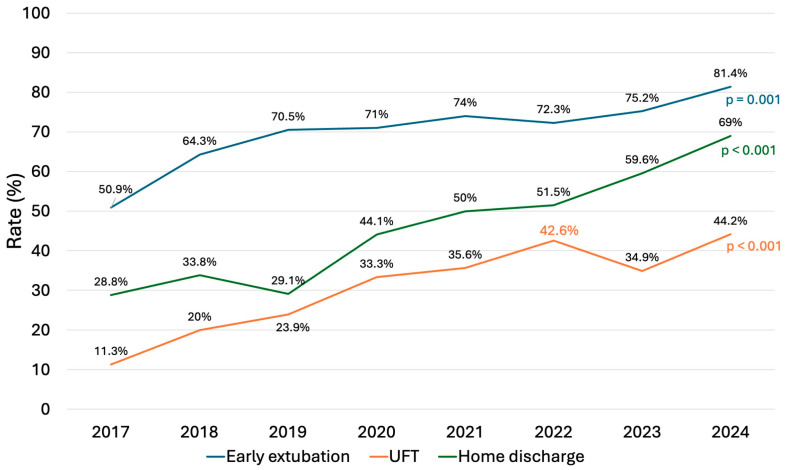
Trends of early extubation (<6 h), ultra-fast-track extubation (on the table), and home discharge over the study period.

**Figure 4:**
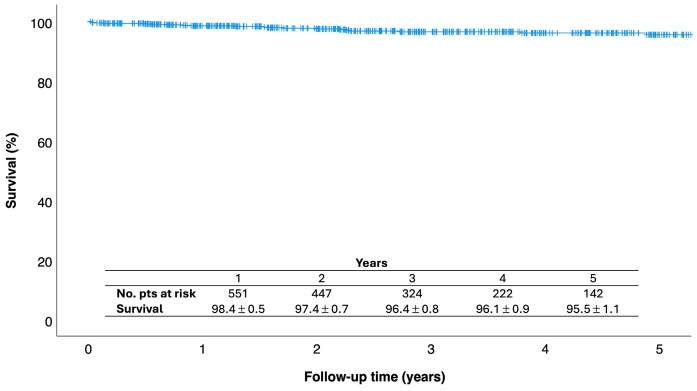
Kaplan–Meier estimates of survival.

In-hospital results are presented in Table 5. The in-hospital mortality rate was 0.6% (n = 4), with a stroke rate of 0.3% (n = 2). The most common postoperative complication was atrial fibrillation in 26% of cases, followed by permanent pacemaker implantation (4.6%), bleeding (3.8%), and acute kidney injury (3.6%), with 0.6% of patients requiring dialysis. Prolonged ventilation (>24 h) was necessary in 17 patients (2.5%). The median intubation time was 4 h (IQR 0–7.5), with 70.8% of patients achieving fast-track extubation (<6 h) and 31.7% achieving ultra-fast-track extubation (on the table). Both fast-track (from 50.9% to 81.4%) and ultra-fast-track (from 11.3% to 44.2%) rates increased considerably over the years (*p* < 0.001) (Figure 3). The median length of stay was 7 days (IQR 6–9), and 46.6% of patients were discharged home directly. Over the study period, there was a significant increase in the proportion of patients discharged home, from 28.8% in 2017 to 69% in 2024 (*p* < 0.001) (Figure 3). Predischarge echocardiography showed mild or less MR in 94.2% (n = 615) of patients, moderate MR in 5.5% (n = 36), and severe MR in 0.3% (n = 2) following reparative surgery. Reoperation for early failure was necessary in seven patients (1%). Re-repair was possible in four patients, with three patients undergoing valve replacement. There were 27 late deaths, and the overall survival at 1-, 3-, and 5 years were 98.4% ± 0.5, 96.4% ± 0.8, and 95.5% ± 1.1, respectively (Figure 4).

## 4. Discussion

This study provides a comprehensive overview of contemporary outcomes in DMR surgery performed at a regional tertiary care center, analyzing a large cohort of patients with complete follow-up, focusing on patient demographics, operative techniques, and early and late results. We believe this analysis is particularly timely, as mitral valve surgery is one of the fastest-growing cardiac procedures captured in national data, with consistently improving outcomes [10,11,12] and due to the increasing adoption of percutaneous techniques for mitral valve repair and replacement. Our findings align with the expanding body of evidence highlighting the ongoing evolution in mitral valve surgery, driven by advancements in minimally invasive techniques, enhanced perioperative care, and improved patient selection criteria. Data from our regional referral center indicate that patients presented for mitral valve interventions are relatively young (66 years) and have low operative risk (ES II 2.4%). Notably, more than four-fifths of DMR patients undergoing surgery still presented with symptoms, while only 13% were referred for surgery without symptoms and with preserved left ventricular function [13]. During the study period, the volume of mitral valve operations increased more than twice (Figure 1), reflecting the growing referral trend for these patients. DMR surgery was confirmed to be associated with excellent results, with very low mortality and morbidity rates and a high survival rate (95.5% at 5 years).

In recent decades, mitral valve surgery has advanced significantly with the introduction of less invasive techniques that better preserve thoracic integrity and minimize surgical trauma [2,4,14,15]. However, despite growing evidence from high-volume specialized centers supporting these less invasive approaches, their widespread adoption in routine clinical practice remains limited [11,16,17]. This is likely due to concerns about increased surgical complexity, a steep learning curve, the need for additional equipment, and higher costs. In our cohort, over 63% of patients underwent a minimally invasive transaxillary approach, with a substantial increase observed over the study period (Figure 2). This finding compares favorably with those reported in many national and international series [11,16,17], suggesting that the transaxillary approach has the potential to expand the adoption of less invasive techniques. As previously shown in a multicenter study [4], transaxillary access is a direct-vision, simplified technique that enhances mitral valve exposure without adding surgical complexity. It minimizes the learning curve, does not require costly video equipment or additional transthoracic instruments, provides outcomes comparable to those of full sternotomy, and improves patient recovery. Our findings confirm these observations, as the increasing use of transaxillary access has also been associated with a higher rate of fast-track recovery and discharge home (Figure 3).

Mitral valve repair was accomplished in 91% of patients despite a significant number having complex valve lesions involving the AML and bileaflet prolapse or flail. When focusing solely on patients with PML lesions, the valve repair rate rose to 93.4%. Furthermore, only 0.9% of patients who initially underwent a repair attempt eventually required valve replacement (Table 3), leading to a 99.1% repair rate among valves that were deemed to be reparable. The repair of AML and bileaflet lesions has traditionally been considered more difficult compared to PML repair, and numerous techniques have been proposed to improve outcomes in patients with such complex valve lesions [14,18]. Notably, in our series, while AML lesion was confirmed to be a negative predictor of successful mitral valve repair, associated with a 2.5-fold higher risk of valve replacement, bileaflet lesion did not emerge as a risk factor for valve replacement and was instead associated with a high repair rate (95.4%). This is likely because the majority of bileaflet lesions occurred in Barlow patients and were corrected using a “simple” annuloplasty-only procedure (88.9%) due to symmetric prolapse with a central regurgitation jet [19,20].

Postoperative SAM is a well-known complication of mitral valve repair surgery, as it can lead to left ventricular outflow tract obstruction and recurrent mitral regurgitation, particularly in patients with specific anatomical features [21]. In our study, postoperative SAM was observed in 1.6% of patients, while 37.6% were identified preoperatively as being at increased risk for SAM. Furthermore, the presence of increased SAM risk factors did not affect the likelihood of successful valve repair. Thus, when SAM is anticipated and appropriately addressed during surgery, good outcomes can be achieved. In such cases, our strategy included careful preoperative echocardiographic assessment, tailored surgical techniques to reduce PML height and shift the coaptation line posteriorly, and proper medical management with intravascular volume expansion, beta-adrenoceptor blockade, and avoidance of inotropic support.

### Limitations

This study has several limitations inherent to its single-center, retrospective design. The choice of surgical technique and the use of minimally invasive approaches were determined by the individual surgeon. Additionally, the study population was drawn from a tertiary referral center, which may limit the generalizability of our findings to patients treated and followed at non-tertiary centers. Finally, no long-term echocardiographic data were collected.

## 5. Conclusions

This study confirms that contemporary surgical techniques for DMR yield excellent early and late outcomes in high-volume heart valve centers. Advanced repair techniques, combined with the increasing use of minimally invasive approaches, have resulted in a high likelihood of valve repair and faster patient recovery. As transcatheter options continue to evolve, these findings reinforce surgical repair as the gold standard treatment for most patients with DMR. We anticipate that the future of mitral valve surgery will involve a nuanced patient selection process that considers factors such as the complexity of mitral pathology, anatomical suitability, and the patient’s overall health profile, alongside the evolving capabilities of transcatheter technologies.

## Figures and Tables

**Table 1:** Demographics.

	N	%
Age, median (IQR)	66	57–74
Male	449	64.9
NYHA		
*I*	90	13.5
*II*	357	53.6
*III*	213	32
*IV*	6	0.9
Hypertension	400	58
Diabetes	37	5.4
Obesity (BMI > 30)	62	9
Preoperative AF	164	23.9
Chronic lung disease	28	4.1
Pacemaker	12	1.7
Dialysis	2	0.3
CAD	40	5.8
Cerebrovascular arteriopathy	14	2
Peripheral arteriopathy	10	1.4
Frailty	19	2.7
Reduced LVEF (<50%)	38	5.5
Euroscore II (median, IQR)	2.4	1.0–4.9

AF: atrial fibrillation. BMI: body mass index. CAD: coronary artery disease. IQR: interquartile range. LVEF: left ventricular ejection fraction. NYHA: New York Heart Association.

**Table 2:** Preoperative echo data.

	N	%
Degenerative type		
*Barlow*	199	28.7
*FED*	299	43.1
*Form fruste*	195	28.1
Lesion site		
*PML*	434	62.6
*AML*	52	7.5
*Bileaflet*	166	24
Flail	393	
*PML*	355	51.4
*AML*	33	4.8
*Bileaflet*	5	0.7
Prolapse	578	
*PML*	374	54.2
*AML*	51	7.4
*Bileaflet*	153	22.2
Commissural prolapse	46	6.7
Annular dilatation	578	85.1
Increased risk for SAM	236	37.6
Pulmonary hypertension (>30 mmHg)	240	36.8
Tricuspid regurgitation		
*No*	312	45.6
*Mild*	312	45.6
*Moderate*	131	19.2
*Severe*	30	4.4
Tricuspid annular dilatation	151	21.8

AML: anterior mitral leaflet. FED: Fibroelastic deficiency. PML: posterior mitral leaflet. SAM: systolic anterior motion.

**Table 3:** Operative data.

	N	%
Surgical access		
*Full sternotomy*	255	36.8
*Minimally invasive*	438	63.2
Conversion to full sternotomy	6	0.9
Concomitant Procedure		
*Tricuspid Surgery*	90	13.1
*AF surgery*	17	2.5
*LAA closure*	36	5.3
*PFO/ASD closure*	12	1.7
Type of surgery		
*Repair*	630	90.9
*Replacement*	56	8.1
*Replacement due to unsuccessful repair*	6	0.9
Repair techniques		
*Artificial chords*	364	54.1
*PML*	316	47
*AML*	43	6.4
*Bileaflet*	5	0.7
*Resection*	119	17.7
*PML*	113	16.8
*AML*	6	0.9
*Sliding plasty*	32	4.8
*Edge to edge*	90	13.4
*Annuloplasty ring*	626	90.3
*Cleft closure*	49	7.1
Cardioplegia type		
*Blood*	207	29.9
*Crystalloid*	484	69.9
Repeated x-clamping due to unsuccessful valve repair	27	3.9
CPB time (min), median (IQR)	92	76–116
Cross-clamp time (min), median (IQR)	62	50–77
Technical success	664	97.5

AF: atrial fibrillation. AML: anterior mitral leaflet. ASD: atrial septal defect. CPB: cardiopulmonary bypass. IQR: interquartile range. LAA: left atrial appendage. PFO: patent foramen ovale. PML: posterior mitral leaflet.

**Table 4:** Predictors of mitral valve replacement.

Variable	*p* Value	OR	95% CI
Age	<0.001	1.053	1.025–1.081
NYHA III-IV	0.07	1.633	0.956–2.791
Lesion site			
*PML (reference)*	*-*	*-*	*-*
*AML*	*0.02*	*2.569*	*1.182–5.585*
*Bileaflet*	*0.6*	*0.851*	*0.399–1.811*
Annulus dilatation	0.6	1.232	0.575–2.614
Commissural lesion	0.3	0.501	0.139–1.807
Prolapse	0.2	0.617	0.277–1.374
Flail	0.9	0.951	0.521–1.735
Increased risk for SAM	0.9	0.998	0.577–1.725

AML: anterior mitral leaflet. CI: confidence interval. NYHA: New York Heart Association. OR: odds ratio. PML: posterior mitral leaflet. SAM: systolic anterior motion.

**Table 5:** In-hospital results.

	N	%
In-hospital mortality	4	0.6
Stroke	2	0.3
Intubation time (hours), median (IQR)	4	0–7.5
Early extubation (<6 h)	482	70.8
Ultra-fast-track (on table extubation)	216	31.7
Ventilation > 24 h	17	2.5
Bleeding (requiring revision)	26	3.8
New onset AF	180	26
Definitive PM	32	4.6
Myocardial infarction	12	1.7
Low cardiac output	19	2.8
Acute kidney injury	25	3.6
Dialysis	4	0.6
Transfusions unit, median (IQR)	0	0–0
Thoracic wound complications	12	1.7
Residual MR post-repair		
*None/trivial*	487	74.6
*Mild*	128	19.6
*Moderate*	36	5.5
*Severe*	2	0.3
Post-operative SAM	11	1.6
Redo for early failure	7	1
ICU stay (hours), median (IQR)	24	22–47.5
Hospital stays (days), median (IQR)	7	6–9
Discharged home	318	46.6

AF: atrial fibrillation. ICU: intensive care unit. IQR: interquartile range. MR: mitral regurgitation. PM: pacemaker.

## Data Availability

The raw data supporting the conclusions of this article will be made available by the authors upon request.
